# Psychological well-being of teachers: influence of burnout, personality, and psychosocial climate

**DOI:** 10.3389/fpsyg.2023.1211280

**Published:** 2023-11-23

**Authors:** Ivette Margarita Espinoza-Díaz, Jordi Tous-Pallarés, Susana Lucas-Mangas, Lorena Valdivieso-León, Andreu Vigil-Colet

**Affiliations:** ^1^Department of Psychology, University of Rovira i Virgilli, Tarragona, Spain; ^2^Department of Psychology, University of Valladolid, Valladolid, Spain

**Keywords:** psychological well-being, burnout, psychological climate, teachers, personality

## Abstract

**Introduction:**

Those who are professionally dedicated to teaching can be exposed with their work to situations that influence their perception of psychological well-being. This study aims to evaluate how the factors of personality, emotional intelligence, burnout and the psychosocial climate derived from the work environment of teachers influence their levels of psychological well-being, to verify whether these variables allow us to establish a predictive model of psychological well-being by means of multiple regression analysis.

**Methods:**

Participants were a group of 386 teachers in early childhood, Primary and Secondary education, both in training and in active service (71.5% women; 28.5% men). A correlation and multiple regression analysis were performed to establish a predictive model of psychological well-being. We used 5 instruments: Psychosocial Climate at Work Scales (ECPT); verall Personality Assessment Scale (OPERAS); Questionnaire for Evaluation of Burnout Syndrome at Work (CESQT); Spanish adaptation of the Riff Psychological Well-being Scales (EBP) and Spanish validation of the Trait Meta-Mood Scale (TMMS-24).

**Results:**

Most of the relationships were significant, and the multiple regression analysis explains 58.5% of the global variance of psychological well-being in teachers, being emotional stability the most relevant and main predictor of psychological well-being, explaining its 38.1%.

**Discussion:**

Personality shows a great influence in psychological well-being of teachers, particularly emotional stability. The ability to establish predictive models to explain psychological well-being in educational environments is confirmed.

## 1 Introduction

Well-being arises from the mediation between health and sickness and is determined by environmental variables, which are regulated by personality (Gale et al., 2013). It can be hedonic, which is characterized by positive affection and the lack of negative affection, or eudaimonic or psychological, which involves the complete psychological functioning of the individual and develops all of their capacities (Millán et al., 2017). Some authors associate the term “psychological well-being” with happiness, satisfaction, subjective well-being, and quality of life (Sánchez-Vidal, 2017; Muñoz-Campos et al., 2018; Lucas-Mangas, 2020).

In recent years, one of the most promising lines of research on work and the mental health of teachers has been the search for explanatory models of well-being based on psychological aspects of work (Aguirre et al., 2020; Orozco et al., 2020). The psychological well-being of these professionals has been shown to be closely related to positive affection, satisfaction, professional success, and good interpersonal relations (Lima and Lerrechea, 2013; Luhmann et al., 2013); high levels of emotional intelligence (Sánchez-Teruel and Robles-Bello, 2018; Aparisi et al., 2019); and person–environment adjustment and perceived social support (Lorente et al., 2008; Martínez, 2020). In this sense, emotional intelligence allows the identification and expression of emotions in an appropriate way, facilitating the understanding and reasoning of one’s own emotions and those of others (Mayer et al., 2000).

In this context, people’s individual resources are one significant element of the construct of well-being that reflects the extent to which they feel that their life is good (Rodríguez-Carvajal et al., 2010). Some authors suggest that one of the main predictors of well-being is personality (Aghababaei and Arji, 2014). However, in organizations, several intertwined variables intervene (Delhom et al., 2019), that can have a positive or negative effect on individuals and, therefore, influence their well-being (Adina and Clipa, 2012).

For this reason, determining the extent to which personal factors and psychological factors of the work environment influence well-being has been controversial: Individual characteristics may affect how people perceive the psychosocial environment at work and consequentially determine their reactions (Díaz-Pincheira and Carrasco-Garcés, 2018) because the psychological environment at work can affect the worker’s well-being, and physical, psychological, and social health (Magnano et al., 2020).

Several studies have addressed this issue, and they confirm that psychological well-being is affected by stressful conditions at work which, if maintained over a long period, can cause emotional discomfort and dissatisfaction which, in turn, lead to burnout (Gil-Monte, 2011; Fiorilli et al., 2019). Burnout is “a process that develops progressively due to the use of poorly functional coping strategies with which professionals try to protect themselves from work stress generated by their relationships with the organization’s clients and, to a lesser extent by their relationships with the organization” (Gil-Monte, 2011, p. 13).

In this regard, Gale et al. (2013) and Larsen (2000) argued that low scores on neuroticism and high scores on extraversion tend to report greater well-being. Since they are related to behaviors associated with psychological well-being, such as occupational achievement and the degree of participation in the community, in this sense, also agreeableness and responsibility are related to greater emotional regulation in terms of interpersonal relationships and adjustment at work. Espinoza-Díaz et al. (2015) working on the same lines found that both personality factors and psychosocial environment greatly influence teachers’ well-being and that disorganization and emotional stability can cause the emergence of burnout syndrome, which affects levels of experienced well-being. In this study, also was found the personality component considered most relevant is “emotional stability.”

Individual differences due to personality play an important role in the evaluation of experienced well-being since individuals can react to the same situation in different ways even though they have the same job. Similarly, personality can moderate how individuals perceive the psychosocial climate at work, definido como se refiere a las condiciones del entorno laboral que pueden afectar el bienestar y la salud de los trabajadores y están directamente relacionadas con la organización en cualquiera de sus tres aspectos: físico, social o psíquico (Tous-Pallarès et al., 2011), that can generate stress (Unda et al., 2016).

Some studies specifically associate emotional stability and extraversion factors with the proneness of experiencing positive and negative emotions, respectively (Diener and Lucas, 1999; Chico, 2000). In this respect, personality benefits psychological well-being in environmental situations that can cause stress and burnout (Durán et al., 2006; Gil-Monte et al., 2009; Queirós et al., 2013), and in turn, this represents a resource that allows identifying the use of different coping strategies (Valdivieso-León et al., 2020).

Therefore, we aimed to evaluate the relationship between personality factors, psychosocial work climate, emotional intelligence, and burnout, on the one hand, the psychological well-being of a group of teachers, on the other hand, determine the extent to which these variables can be used to create a predictive model of psychological well-being using multiple regression analysis. Personality factors are thought to have a special influence on the perception of the psychosocial climate leading to burnout and on the person–environment adjustment that favors a greater sense of well-being experienced by teachers (Moreno-Jiménez et al., 2012; Merino-Tejedor and Lucas-Mangas, 2016; Soler et al., 2016; De la Fuente et al., 2020).

## 2 Materials and methods

### 2.1 Participants

The participants in the study were 386, both in training and in active service, of whom, 71.5% were women and 28.5% were men, aged between 18 and 64 years (x̄ = 31; SD = 10.94). The study was carried out with a non-probabilistic sample, the response rate of which was 99%. The teachers were working in early childhood (23.6%), primary (42.5%), and secondary (33.9%) education, and 76.7% of them were full-time, 11.1% were part-time, and 9.3% were employed by the hour. The remaining 2.8% did not report information in this regard.

### 2.2 Instruments

In order to ensure that the variables being studied were operative, the following five instruments were used:

- Scales of Psychosocial Climate at Work (ECPT) by Tous-Pallarès et al. (2011). This brief questionnaire diagnoses the psychosocial factors that workers perceive as negative or positive in their organizations. It consists of 16 items and three scales: work content (WC; α = 0.95), personal relationships (PR α = 0.90), and role definition (disorganization) (DR; α = 0.89).
- Overall Personality Assessment Scale (OPERAS) by Vigil-Colet et al. (2013). This is a 40-item scale based on the model of the five personality factors, and the scores provided are free from the effects of acquiescence and social desirability. The factorial reliability of the scales is the following: extraversion (EX; α = 0.86), responsibility (RE; α = 0.77), emotional stability (ES; α = 0.86), kindness (K; α = 0.71), and openness to experience (OE; α = 0.81).
- Questionnaire for the Evaluation of Burnout Syndrome at Work (CESQT) by Gil-Monte (2011). This questionnaire evaluates cognitions, emotions, and attitudes related to work experiences. It consists of 20 items and covers four dimensions: illusion for work (IW; α = 0.90), mental exhaustion (ME; α = 0.85), indolence (IN; α = 0.74), guilt (G; α = 0.82), as well as a general burnout scale (GBS; α = 0.85).
- Spanish adaptation of Riff‘s Psychological Well-being Scales (EBP) by Díaz et al. (2006). This is a reduced scale of 29 items, which measures six dimensions of psychological well-being: self-acceptance (SA; α = 0.84), positive relationships (PR; α = 0.78), autonomy (AT; α = 0.70), domain of the environment (DE; α = 0.82), purpose in life (PL; α = 0.70), and personal growth (PG; α = 0.71).
- Spanish validation of the Trait Meta-Mood Scale-24 (TMMS-24) by Fernández-Berrocal et al. (2004). This is a scale of 24 items and three emotional intelligence dimensions: attention (AT; α = 0.90), clarity (CL; α = 0.90), and emotional repair (ER; α = 0.86).

### 2.3 Procedure

We contacted the Ministry of Education of Catalunya to arrange a meeting with the directors of the schools in the region so that we could explain the project, sign agreements with those schools interested (a total of 5), and subsequently apply the battery of tests to their teachers. They were provided with information on the objectives of the psychosocial evaluation, with particular emphasis on the importance of giving honest answers to the questionnaires. Similarly, they were told that the agreement with the school included a confidentiality agreement that ensured that the information provided would be properly processed and used. This was explained on the forms used to administer the tests.

The test was administered through a web platform hosted on our laboratory servers. Our team contacted the participants, and we spread the link through which they could access the platform hosting the test, where they could leave their answers. The tests were administered on Spanish language. The platform had a filter that guaranteed that the participants voluntarily agreed to be part of the study, without any type of coercion or financial remuneration, and participants also stated that they had been informed that the administration of the scale was completely anonymous and governed by the Data Protection Act.

The research was also authorized by the Ethics Committee of University of Rovira i Virgilli with protocol code CEIPSA-2021-PR-0056.

### 2.4 Data analysis

The test scores were collected in a database with the program SPSS version 26, and subsequently, a correlation and multiple regression analysis was performed in successive steps in order to establish the influence of the study variables and the relationship between them.

## 3 Results

The results showed that most of the relationships obtained from the study were significant. It can be seen in Table 1 that the variables analyzed—except the professional life stage—show substantial relationships with the measures of psychological well-being. However, the relationship was greater between personality measures and the total sum of psychological well-being, especially emotional stability (*r* = 0.617; *p* < 0.01), and to a lesser extent, the relationships obtained with emotional intelligence are also remarkable in their emotional repair scale (*r* = 0.523; *p* < 0.01), with the total burnout scale (*r* = −0.498; *p* < 0.01) and with psychosocial climate with their personal relationships scale (*r* = 0.379; *p* < 0.01).

**Table 1:** Pearson correlations between the variables of psychosocial climate, personality, emotional intelligence, professional life stage, and burnout with the psychological well-being of teachers.

Pearson correlations	SA	PR	AT	DE	PG	PL	TPW
**Psychosocial climate**							
Job content	**0.253**	**0.211**	*0.107*	**0.331**	**0.150**	**0.277**	**0.297**
Personal relationships	**0.327**	**0.351**	*0.105*	**0.315**	**0.248**	**0.347**	**0.379**
Definition of role (disorganization)	**−0.168**	**−0.225**	−0.046	**−0.276**	**−0.147**	**−0.190**	**−0.239**
**Personality**							
Extraversion	**0.330**	**0.362**	**0.251**	**0.256**	**0.171**	**0.304**	**0.370**
Emotional stability	**0.603**	**0.431**	**0.426**	**0.526**	**0.333**	**0.511**	**0.617**
Responsibility	**0.209**	**0.161**	0.097	**0.274**	**0.228**	**0.318**	**0.288**
Kindness	**0.272**	**0.291**	0.095	**0.288**	**0.264**	**0.217**	**0.319**
Openness to experience	**0.206**	**0.174**	**0.193**	**0.267**	**0.294**	**0.240**	**0.294**
**Emotional intelligence**							
Emotional attention	**−0.165**	0.061	**−0.251**	*−0.127*	0.076	−0.029	−0.087
Emotional clarity	**0.383**	**0.291**	**0.280**	**0.399**	**0.337**	**0.426**	**0.455**
Emotional repair	**0.535**	**0.320**	**0.328**	**0.371**	**0.412**	**0.421**	**0.523**
**Burnout**							
Illusion for Work	**0.302**	**0.390**	*0.119*	**0.333**	**0.347**	**0.374**	**0.414**
Psychological tiredness	**−0.259**	**−0.348**	**−0.184**	**−0.330**	**−0.179**	**−0.275**	**−0.356**
Indolence, cynicism	**−0.265**	**−0.301**	**−0.221**	**−0.377**	**−0.256**	**−0.292**	**−0.376**
Guilt	**−0.248**	**−0.240**	**−0.260**	**−0.315**	**−0.205**	**−0.256**	**−0.328**
Burnout total scale	**−0.359**	**−0.454**	**−0.232**	**−0.454**	**−0.330**	**−0.406**	**−0.498**
Professional life stage	0.035	*−0.114*	*0.107*	0.057	−0.034	0.018	0.002

Significant values: *p* < 0.01 in **bold**, *p* < 0.05 in *italics*; SA, self-acceptance; PR, positive relationships; AT, autonomy; DE, domain of environment; PG, personal growth; PL, purpose in life; TPW, total psychological well-being.

These results indicate that emotional stability is the personality factor that influences psychological well-being of teachers the most, especially self-acceptance and domain of the environment. However, the emotional repair scale is also noteworthy on self-acceptance and personal growth. The total burnout scale had the most effect on positive relationships and the domain of the environment. The psychosocial climate scale intervenes in the ability to establish positive relationships and the purpose of life. Therefore, teachers with greater emotional stability, who are able to repair their emotions and who perceive good personal relationships in their work environment minimize the effects of burnout. This enables them to master the environment and interact in a positive way, which in turn influences their psychological well-being.

The next step consisted of carrying out a series of multiple regressions in successive steps to establish a predictive model of the psychological well-being of teachers. In these analyses, the total score of psychological well-being and the scores of the scales that make it up were used as criteria as were the scores of the predictive scales psychosocial climate, personality, emotional intelligence, and burnout, and the variable stage of professional life.

Table 2 shows the results obtained from the analysis. The best predictor of the total scale of psychological well-being is emotional stability, which explains more than 38% of the variance and, with the personality factors, a total of 40.4%. The dimensions of burnout, especially its total scale, increase by 8.7%, while emotional intelligence in its dimension of emotional clarity contributes a total of 6%. The psychosocial climate with its scale of personal relationships increases by 1.3%, making the total variance explained by the model a total of 58.5%. It should be pointed out that the stage of professional life variable did not enter the model at any time.

**Table 2:** Predictive model successive steps of the psychological well-being of teachers through the analysis of personality factors, psychosocial climate, emotional intelligence, professional life stage, and burnout.

Regression analysis	SA		RL		AT		DE		PG		PL	
	*R* ^2^	β	*R* ^2^	β	*R* ^2^	β	*R* ^2^	β	*R* ^2^	β	*R* ^2^	β
**Psychosocial climate**												
Job content	–	–	–	–	–	–	0.022	0.147	–	–	–	–
Personal relationships	0.019	0.139	0.029	0.150	-	-	-	-	-	-	0.010	0.126
Definition of role (disorganization)	–	–	–	–	–	–	–	–	–	–	–	–
**Personality**												
Extraversion	0.006	0.090	0.021	0.142	0.013	0.126	–	–	–	–	–	–
Emotional stability	0.364	0.402	0.078	0.194	0.181	0.251	0.227	0.247	0.020	0.140	0.261	0.327
Responsibility	–	–	–	–	–	–	0.009	0.106	–	–	0.025	0.155
Kindness	–	–	–	–	0.014	−0.138	–	–	–	–	0.009	−0.107
Openness to experience	–	–	–	–	0.012	0.114	0.015	0.106	0.045	0.192	–	–
**Emotional intelligence**												
Emotional attention	0.009	−0.120	–	–	0.022	−0.246	0.010	−0.119	–	–	–	–
Emotional clarity	0.036	0.208	0.010	0.108	0.038	0.197	0.048	0.251	0.080	0.216	0.072	0.249
Emotional repair	–	–	–	–	–	–	–	–	–	–	–	–
**Burnout**												
Illusion for work	–	–	–	–	–	–	–	–	0.120	0.205	0.047	0.134
Psychological tiredness	–	–	–	–	–	–	–	–	–	–	–	–
Indolence, cynicism	0.015	−0.118	–	–	0.016	−0.148	0.008	−0.136	0.010	−0.111	0.013	−0.146
Guilt	–	–	–	–	–	–	–	–	–	–	–	–
Burnout total scale	–	–	0.206	−0.275	–	–	0.076	−0.139	–	–	–	–
Professional life stage	–	–	–	–	–	–	–	–	–	–	–	–
Explained variance	**44.9**		**33.4**		**29.7**		**46.5**		**27.5**		**43.6**	

*p* < 0.01; SA, self acceptance; RL, positive relationships; AT, autonomy; DE, domain of environment; PG, personal growth; PV, purpose in life. Bold aims to highlight the variance explained.

In the results obtained for each of the psychological well-being scales, the highest percentage of variance is constantly explained by emotional stability or burnout. The proposed model explains 44.9% of the variance of self-acceptance, 33.4% of positive relationships, 29.7% of personal autonomy, 46.5% of the domain of the environment, 27.5% of personal growth, and 43.6% of the purpose in life (see Table 3).

**Table 3:** Predictive model successive steps of the total psychological well-being of teachers through the analysis of personality factors, psychosocial climate, emotional intelligence, professional life stage, and burnout (total explained variance of psychological well-being).

Regression analysis	TPW	
	*R* ^2^	*β*
**Psychosocial climate**		
Job content	–	–
Personal relationships	0.014	0.116
Definition of role (disorganization)	**–**	**–**
**Personality**		
Emotional stability	0.381	0.383
Extraversion	**–**	**–**
Responsibility	0.005	0.080
Kindness	**–**	**–**
Openness to experience	0.018	0.134
**Emotional intelligence**		
Emotional attention	–	–
Emotional clarity	0.060	0.236
Emotional repair	0.026	0.193
**Burnout**		
Illusion for work	–	–
Psychological tiredness	–	–
Indolence, cynicism	0.081	−0.374
Guilt	–	–
Professional life stage	–	–
Total explained variance	**58.5%**	

*p* < 0.01; TPW, total psychological well-being. Bold aims to highlight the variance explained.

These results show that personality factors, and particularly emotional stability, have the greatest power to predict psychological well-being in teachers although burnout scales, together with, emotional intelligence and psychosocial climate, also play a fundamental role in increasing this predictive capacity.

## 4 Discussion

This study shows important relationships between personality factors and all dimensions of psychological well-being, especially emotional stability (*r* = 0.617; *p* < 0.01) and extraversion (*r* = 0.370; *p* <. 01), similar to study by Rakesh (2011) who finds a similar relationship between emotional stability (*r* = 0.490; *p* < 0.01) and extraversion (*r* = 0.260; *p* < 0.01). Both studies coincide with those by other authors who show the same relationship (Chico, 2000; Gale et al., 2013) as well as significant relationships between all the personality factors and satisfaction and psychological well-being (Delhom et al., 2019).

For its part, each of the scales of psychosocial climate, especially personal relationships (*r* = 0.379; *p* < 0.01), presents a high correlation with psychological well-being. Faragher et al. (2005) found values ranging from (*r* = 0.235; *p* < 0.01) to (*r* = −0.287; *p* < 0.01) when they correlated job satisfaction with personal relationships. Similarly, the emotional intelligence scales of emotional clarity and repair also show a high correlation with psychological well-being scales. This result is similar to that obtained by Adina and Clipa (2012) when they correlated emotional intelligence with positive attitudes at work (*r* = 0.417; *p* < 0.01), job satisfaction (*r* = 0.255; *p* < 0.01), and general satisfaction in life (*r* = 0.229; *p* < 0.01).

High correlations (*r* = 0.328 to *r* = 0.498) are observed between burnout and all scales of psychological well-being. This result concurs with that of Faragher et al. (2005), who correlated job satisfaction and burnout with similar results (*r* = 0.409 to *r* = 0.478).

**Figure 1:**
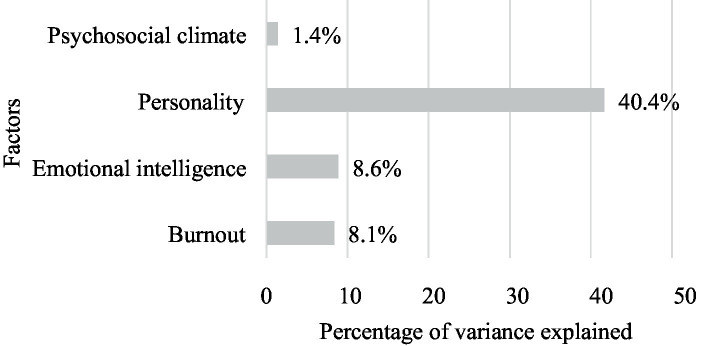
Explanatory model of psychological well-being in teachers with the variables of personality, emotional intelligence, burnout, and psychosocial climate. Total explained variance of psychological well-being 58.5%.

Moreover, the results of the multiple regression analysis show that all the personality factors together explain 40.4% of the psychological well-being of teachers. However, the model shows that emotional stability is the main predictor of psychological well-being, explaining 38.1% of its variance, in agreement with Chico (2000), who found that emotional stability explained 44% of psychological well-being, and Rakesh (2011), who found that personality factors explained 21%. Second, as can be seen in Figure 1, the model introduces the total burnout scale, which explains 8.1% of the total variance in psychological well-being. This result is similar to that of Durán et al. (2006), who explain 8.2% of satisfaction with burnout and emotional fatigue, and Queirós et al. (2013), who explain 11.4%. Third, emotional intelligence contributes 8.6% to the total variance of the psychological well-being of teachers. In this case, it is interesting to note that the result coincides with the conclusions of study by Aguirre et al. (2020) that when personality is taken into account, emotional intelligence predicts only a small variation in satisfaction and provides much less information than personality factors.

## 5 Conclusion

As a result of the observed results, we can conclude that the presence of burnout in teachers is mainly related to the perception of disorganization in the work environment, which influences the decrease in enthusiasm for it. However, it is also observed that in those teachers who are exposed to burnout but who present greater emotional stability and kindness, it is more likely that it does not affect them; that is, the stress derived from work favors the presence of burnout, and it is experienced by the individual. Therefore, it can only be explained with context and personality variables (Moreno-Jiménez et al., 2006, 2012). Therefore, we can affirm that the main predictor of burnout in teachers is the perception of disorganization in the work environment; however, the mediating role played by personality is undeniable, especially emotional stability and extraversion, since both contribute to a greater emotional clarity and ability to repair, Therefore, the most emotionally stable and extroverted teachers minimize the negative effects of environmental factors, decreasing to a certain extent the burnout experience they face, since, as Chico (2000) affirms, people assess quality of their lives according to their own personal criteria.

Therefore, it can be affirmed that psychological well-being is influenced by personality (particularly by emotional stability); however, emotional intelligence and SQT also present a relevant relationship. However, the emotional stability personality factor is the main predictor of psychological well-being and the use of measures of SQT, psychosocial climate, and EI increase the predictive capacity of the model, and the joint use of measures of psychosocial climate and personality improves the ability to establish predictive models that allow the prevention of burnout as well as improve psychological well-being in educational environments with respect to models that focus exclusively on environmental variables or on individual variables.

Finally, the model introduces the scale of psychosocial climate and personal relationships, which explains a small percentage but not less than 1.4% of the variance since relationships with others constitute the basis for the emergence of physical and mental health problems (Lima and Lerrechea, 2013; Millán et al., 2017).

It is concluded that psychological well-being is influenced by personality (particularly by emotional stability) although emotional intelligence and burnout also have an effect. The personality factor emotional stability, then, is the main predictor of psychological well-being in teachers and the measures of burnout, psychosocial climate, and emotional intelligence increase the predictive capacity of the model, which explains 56.6% of the overall variance of the study (Lucas-Mangas, 2020). Therefore, the joint use of Psychosocial Climate and Models that include personality measures can predict and prevent burnout and improve psychological well-being in teaching environments more effectively than models that focus exclusively on environmental or individual variables.

After analyzing the results, this study presents an important practical implication in the initial evaluation of teacher candidates since if they are emotionally stable it will have an impact on their emotional well-being when they are in active service. It is suggested to use the tests that are used to identify burnout and well-being levels in teachers to develop preventive detection actions.

Finally, the main limitation of this research is that a larger sample would be more representative and would make it possible to provide a model that is more representative of the Spanish population; it is an ambitious project on which work continues. It would be interesting to conduct a longitudinal study to identify the changes that could occur among the factors involved and their impact on burnout and psychological well-being.

## Data availability statement

The raw data supporting the conclusions of this article will be made available by the authors, without undue reservation.

## Ethics statement

The studies involving humans were approved by Ethics Committee of University of Rovira i Virgili. The studies were conducted in accordance with the local legislation and institutional requirements. The participants provided their written informed consent to participate in this study.

## Author contributions

SL-M, JT-P, and IE-D: conceptualization. IE-D, AV, and JT-P: methodology. JT-P and IE-D: formal analysis and supervision. SL-M, LV-L, JT-P, and IE-D: investigation, resources, and writing – original draft preparation. IE-D and AV: data curation. SL-M, LV-L, JT-P, AV, and IE-D: writing – review and editing. LV-L: visualization. All authors contributed to the article and approved the submitted version.
